# Supplementary material for: Transcriptomic Analysis of Mineralized Adipose-Derived Stem Cell Tissues for Calcific Valve Disease Modelling
Source: Int J Mol Sci. 2024 Feb 14;25(4):2291. doi: 10.3390/ijms25042291 (PMC10889332; doi:10.3390/ijms25042291)
Supplement: Supplementary file 1 [file ijms-25-02291-s001.zip › Supplementary Figure S1.pdf]

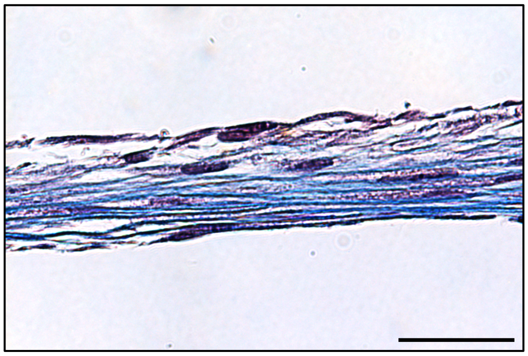

Supplementary Figure S1. Histological staining of uninduced 3D ASC-sheet produced by the self-assembly method of tissue engineering. Masson trichrome staining of an ASC-sheet horizontal section showing collagen marked in blue, nuclei in dark red/purple, and cytoplasm stained in pink/light purple. Microscopic images were taken with Zeiss Axio Imager M2 microscope with an AxioCam ICc1 camera (Zeiss Canada Ltd., Toronto, ON, Canada). Scale bar = 50μm.
