# Supplementary material for: Transcriptomic Analysis of Mineralized Adipose-Derived Stem Cell Tissues for Calcific Valve Disease Modelling
Source: Int J Mol Sci. 2024 Feb 14;25(4):2291. doi: 10.3390/ijms25042291 (PMC10889332; doi:10.3390/ijms25042291)
Supplement: Supplementary file 1 [file Supplementary Figure S2.pdf › Supplementary Figure S2.pdf]

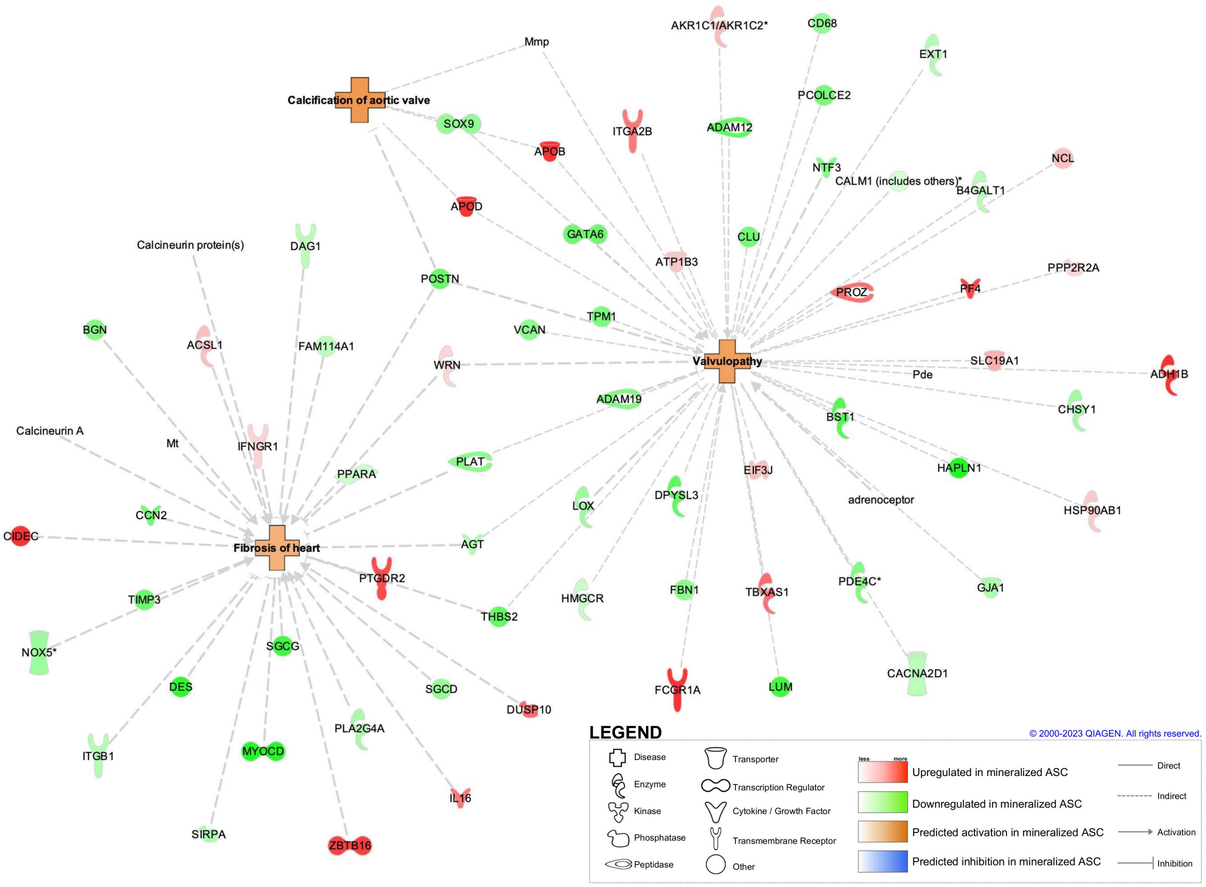

© 2000-2023 QIAGEN. All rights reserved.

Supplementary figure S2. ***In silico* prediction of valve disease.** IPA generated interactome of modulated genes linked to various valve diseases (valvulopathy, fibrosis of heart and calcification of aortic valve).
